# Obesity research: Moving from bench to bedside to population

**DOI:** 10.1371/journal.pbio.3002448

**Published:** 2023-12-04

**Authors:** Ann Marie Schmidt

**Affiliations:** Diabetes Research Program, Department of Medicine, New York University Grossman School of Medicine, New York, New York, United States of America

## Abstract

Globally, obesity is on the rise. This Perspective looks at how research over the past 20 years has changed the way we view obesity and what the future might hold for further mechanistic understanding and treatment and prevention options.

## Teaser

Globally, obesity is on the rise. Research over the past 20 years has highlighted the far-reaching multisystem complications of obesity, but a better understanding of its complex pathogenesis is needed to identify safe and lasting solutions.

This article is part of the *PLOS Biology* 20th anniversary collection.

Obesity is a multifaceted disorder, affecting individuals across their life span, with increased prevalence in persons from underrepresented groups. The complexity of obesity is underscored by the multiple hypotheses proposed to pinpoint its seminal mechanisms, such as the “energy balance” hypothesis and the “carbohydrate–insulin” model. It is generally accepted that host (including genetic factors)–environment interactions have critical roles in this disease. The recently framed “fructose survival hypothesis” proposes that high-fructose corn syrup (HFCS), through reduction in the cellular content of ATP, stimulates glycolysis and reduces mitochondrial oxidative phosphorylation, processes that stimulate hunger, foraging, weight gain, and fat accumulation [1]. The marked upswing in the use of HFCS in beverages and foods, beginning in the 1980s, has coincided with the rising prevalence of obesity.

**Fig 1:**
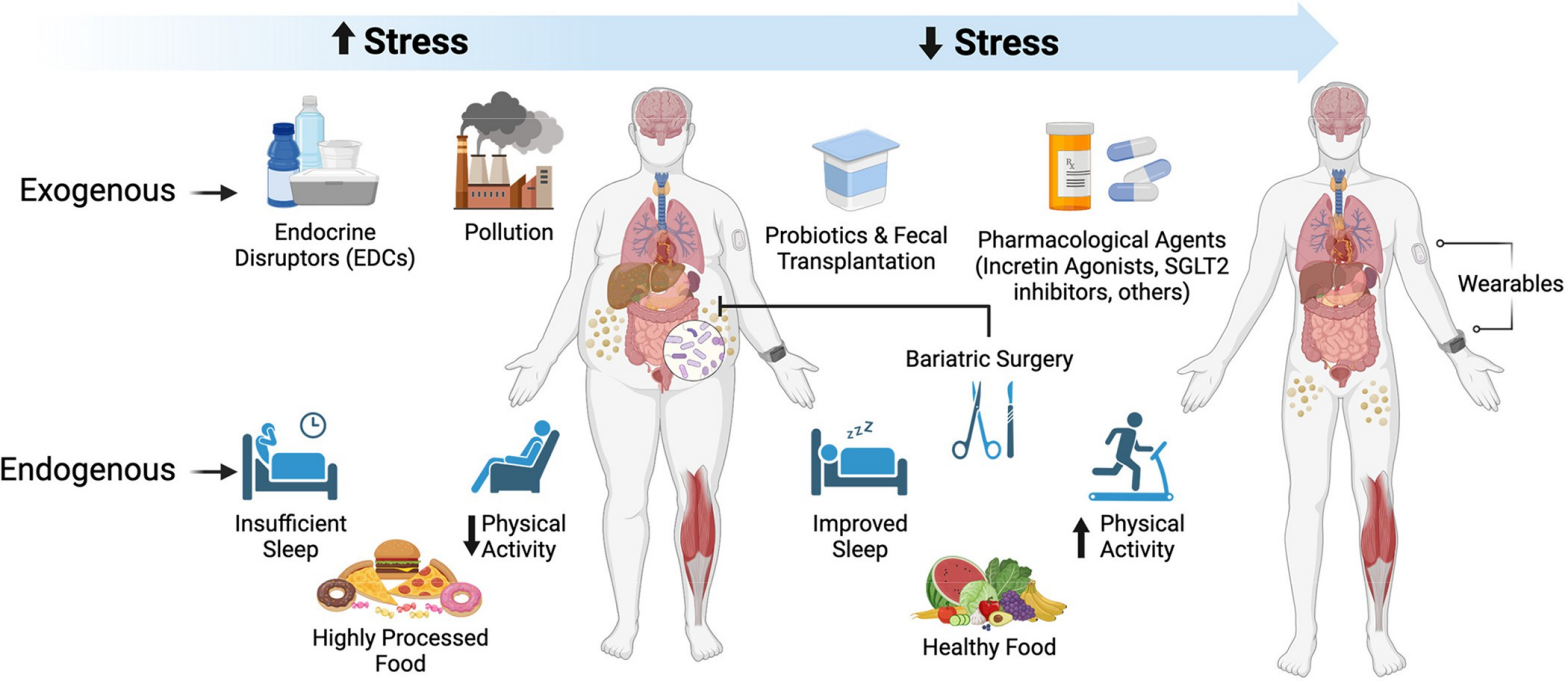
Leaps in the understanding of obesity in the past 20 years: Uncovering new mechanisms and identifying state-of-the-art multidisciplinary treatment approaches. Basic, clinical/translational, and epidemiological research has made great strides in the past few decades in uncovering novel components of cell-intrinsic, intercellular, and interorgan communications that contribute to the pathogenesis of obesity. Both endogenous and exogenous (environmental) stressors contribute to the myriad of metabolic perturbations that impact energy intake and expenditure; mediate innate disturbances in the multiple cell types affected in obesity in metabolic organelles and organs, including in immune cells; and impair beneficial interkingdom interactions of the mammalian host with the gut microbiome. The past few decades have also witnessed remarkable efforts to successfully treat obesity, such as the use of the incretin agonists and bariatric surgery. Yet, these and other strategies may be accompanied by resistance to weight loss, weight regain, adverse effects of interventions, and the challenges of lifelong implementation. Hence, through leveraging novel discoveries from the bench to the bedside to the population, additional strategies to prevent obesity and weight regain post-weight loss, such as the use of “wearables,” with potential for implementation of immediate and personalized behavior modifications, may hold great promise as complementary strategies to prevent and identify lasting treatments for obesity. Figure created with BioRender.

The past few decades of scientific progress have dramatically transformed our understanding of pathogenic mechanisms of obesity (Fig 1). Fundamental roles for inflammation were unveiled by the discovery that tumor necrosis factor-α contributed to insulin resistance and the risk for type 2 diabetes in obesity [2]. Recent work has ascribed contributory roles for multiple immune cell types, such as monocytes/macrophages, neutrophils, T cells, B cells, dendritic cells, and mast cells, in disturbances in glucose and insulin homeostasis in obesity. In the central nervous system, microglia and their interactions with hypothalamic neurons affect food intake, energy expenditure, and insulin sensitivity. In addition to cell-specific contributions of central and peripheral immune cells in obesity, roles for interorgan communication have been described. Extracellular vesicles emitted from immune cells and from adipocytes, as examples, are potent transmitters of obesogenic species that transfer diverse cargo, including microRNAs, proteins, metabolites, lipids, and organelles (such as mitochondria) to distant organs, affecting functions such as insulin sensitivity and, strikingly, cognition, through connections to the brain [3].

Beyond intercellular communication mediated by extracellular vesicles, the discovery of interactions between the host and the gut microbiome has suggested important roles for this interkingdom axis in obesity. Although disturbances in commensal gut microbiota species and their causal links to obesity are still debated, transplantation studies have demonstrated relationships between Firmicutes/Bacteroidetes ratios and obesity [4]. Evidence supports the concept that modulation of gut microbiota phyla modulates fundamental activities, such as thermogenesis and bile acid and lipid metabolism. Furthermore, compelling discoveries during the past few decades have illustrated specific mechanisms within adipocytes that exert profound effects on organismal homeostasis, such as adipose creatine metabolism, transforming growth factor/SMAD signaling, fibrosis [5], hypoxia and angiogenesis, mitochondrial dysfunction, cellular senescence, impairments in autophagy, and modulation of the circadian rhythm. Collectively, these recent discoveries set the stage for the identification of potential new therapeutic approaches in obesity.

Although the above discoveries focus largely on perturbations in energy metabolism (energy intake and expenditure) as drivers of obesity, a recently published study suggests that revisiting the timeline of obesogenic forces in 20th and 21st century society may be required. The authors tracked 320,962 Danish schoolchildren (born during 1930 to 1976) and 205,153 Danish male military conscripts (born during 1939 to 1959). Although the overall trend of the percentiles of the distributions of body mass index were linear across the years of birth, with percentiles below the 75th being nearly stable, those above the 75th percentile demonstrated a steadily steeper rise the more extreme the percentile; this was noted in the schoolchildren and the military conscripts [6]. The authors concluded that the emergence of the obesity epidemic might have preceded the appearance of the factors typically ascribed to mediating the obesogenic transformation of society by several decades. What are these underlying factors and their yet-to-be-discovered mechanisms?

First, in terms of endogenous factors relevant to individuals, stressors such as insufficient sleep and psychosocial stress may impact substrate metabolism, circulating appetite hormones, hunger, satiety, and weight gain [7]. Reduced access to healthy foods rich in vegetables and fruits but easy access to ultraprocessed ingredients in “food deserts” and “food swamps” caused excessive caloric intake and weight gain in clinical studies [8]. Second, exogenous environmental stresses have been associated with obesity. For example, air pollution has been directly linked to adipose tissue dysfunction [9], and ubiquitous endocrine disruptor chemicals (EDCs) such as bisphenols and phthalates (found in many items of daily life including plastics, food, clothing, cosmetics, and paper) are linked to metabolic dysfunction and the development of obesity [10]. Hence, factors specific to individuals and their environment may exacerbate their predisposition to obesity.

In addition to the effects of exposure to endogenous and exogenous stressors on the risk of obesity, transgenerational (passed through generations without direct exposure of stimulant) and intergenerational (direct exposure across generations) transmission of these stressors has also been demonstrated. A leading proposed mechanism is through epigenetic modulation of the genome, which then predisposes affected offspring to exacerbated responses to obesogenic conditions such as diet. A recent study suggested that transmission of disease risk might be mediated through transfer of maternal oocyte-derived dysfunctional mitochondria from mothers with obesity [11]. Additional mechanisms imparting obesogenic “memory” may be evoked through “trained immunity.”

Strikingly, the work of the past few decades has resulted in profound triumphs in the treatment of obesity. Multiple approved glucagon-like peptide 1 (GLP1) and gastric inhibitory polypeptide (GIP) agonists [12] (alone or in combinations) induce highly significant weight loss in persons with obesity [13]. However, adverse effects of these agents, such as pancreatitis and biliary disorders, have been reported [14]. Therefore, the long-term safety and tolerability of these drugs is yet to be determined. In addition to pharmacological agents, bariatric surgery has led to significant weight loss as well. However, efforts to induce weight loss through reduction in caloric intake and increased physical activity, pharmacological approaches, and bariatric surgery may not mediate long-term cures in obesity on account of resistance to weight loss, weight regain, adverse effects of interventions, and the challenges of lifelong implementation of these measures.

Where might efforts in combating obesity lie in the next decades? At the level of basic and translational science, the heterogeneity of metabolic organs could be uncovered through state-of-the-art spatial “omics” and single-cell RNA sequencing approaches. For example, analogous to the deepening understanding of the great diversity in immune cell subsets in homeostasis and disease, adipocyte heterogeneity has also been suggested, which may reflect nuances in pathogenesis and treatment approaches. Further, approaches to bolster brown fat and thermogenesis may offer promise to combat evolutionary forces to hoard and store fat. A better understanding of which interorgan communications may drive obesity will require intensive profiling of extracellular vesicles shed from multiple metabolic organs to identify their cargo and, critically, their destinations. In the three-dimensional space, the generation of organs-on-a-chip may facilitate the discovery of intermetabolic organ communications and their perturbations in the pathogenesis of obesity and the screening of new therapies.

Looking to prevention, recent epidemiological studies suggest that efforts to tackle obesity require intervention at multiple levels. The institution of public health policies to reduce air pollution and the vast employment of EDCs in common household products could impact the obesity epidemic. Where possible, the availability of fresh, healthy foods in lieu of highly processed foods may be of benefit. At the individual level, focused attention on day-to-day behaviors may yield long-term benefit in stemming the tide of obesity. “Wearable” devices that continuously monitor the quantity, timing, and patterns of food intake, physical activity, sleep duration and quality, and glycemic variability might stimulate on-the-spot and personalized behavior modulation to contribute to the prevention of obesity or of maintenance of the weight-reduced state.

Given the involvement of experts with wide-ranging expertise in the science of obesity, from basic science, through clinical/translational research to epidemiology and public health, it is reasonable to anticipate that the work of the next 2 decades will integrate burgeoning multidisciplinary discoveries to drive improved efforts to treat and prevent obesity.

## Abbreviations

EDC: endocrine disruptor chemical
GIP: gastric inhibitory polypeptide
GLP1: glucagon-like peptide 1
HFCS: high-fructose corn syrup
